# Biopolymer Hydrogel Scaffold as an Artificial Cell Niche for Mesenchymal Stem Cells

**DOI:** 10.3390/polym12112550

**Published:** 2020-10-30

**Authors:** Marfa N. Egorikhina, Yulia P. Rubtsova, Irina N. Charykova, Marina L. Bugrova, Irina I. Bronnikova, Polina A. Mukhina, Larisa N. Sosnina, Diana Ya. Aleynik

**Affiliations:** Federal State Budgetary Educational Institution of Higher Education “Privolzhsky Research Medical University” of the Ministry of Health of the Russian Federation, Nizhny Novgorod 603005, Russia; rubincherry@yandex.ru (Y.P.R.); irina-ch0709@yandex.ru (I.N.C.); marysmir@mail.ru (M.L.B.); ira-bronikova-2014@yandex.ru (I.I.B.); polina19951512muhina@mail.ru (P.A.M.); larsos@mail.ru (L.N.S.); daleynik@yandex.ru (D.Y.A.)

**Keywords:** scaffold, hydrogel, biopolymers, fibrinogen, collagen, mesenchymal stem cells, VEGF, structure, proliferation, immunophenotype

## Abstract

The activity of stem cell processes is regulated by internal and external signals of the cell “niche”. In general, the niche of stem cells can be represented as the microenvironment of the cells, providing a signal complex, determining the properties of the cells. At the same time, the “niche” concept implies feedback. Cells can modify their microenvironment, supporting homeostasis or remodeling the composition and structure of the extracellular matrix. To ensure the regenerative potential of tissue engineering products the “niche” concept should be taken into account. To investigate interactions in an experimental niche, an original hydrogel biopolymer scaffold with encapsulated mesenchymal adipose-derived stem cells (ASCs) was used in this study. The scaffold provides for cell adhesion, active cell growth, and proliferative activity. Cells cultured within a scaffold are distinguished by the presence of a developed cytoskeleton and they form a cellular network. ASCs cultured within a scaffold change their microenvironment by secreting VEGF-A and remodeling the scaffold structure. Scaffold biodegradation processes were evaluated after previous culturing of the ASCs in the scaffolds for periods of either 24 h or six days. The revealed differences confirmed that changes had occurred in the properties of scaffolds remodeled by cells during cultivation. The mechanisms of the identified changes and the possibility of considering the presented scaffold as an appropriate artificial niche for ASCs are discussed.

## 1. Introduction

The high regenerative potential of mesenchymal stem cells (MSCs) has made them a mainstream cellular material for the treatment of various diseases and for tissue engineering [1,2,3,4,5]. The FDA has already registered more than 980 studies using MSCs, involving more than 10,000 patients [6,7,8]. However, the study results have not always confirmed the effectiveness of MSCs and results have often fallen short of expectations. What could be the reason? It is known that when moving outside their niche, stem cells can quickly lose their regenerative potential and die, and this severely limits their clinical use. This is often associated with the phenomenon of anoikis (anoikis—from the Greek “homelessness”) [9]. So, in vivo, stem cells regulate their behavior, guided by external signals that they receive from their local niche [10,11]. These signals are extremely diverse in nature: resulting from the local extracellular matrix (ECM), from junctions with neighboring cells, and signals from growth factors, hormones, etc. [12,13]. When MSCs are isolated from their local niche and transplanted, the cells enter an alien microenvironment in which the necessary signals are absent, and which, ultimately, can bring the expected regenerative effect to naught [14]. The solution to the problem is to create an artificial niche, providing a sufficient level of appropriate signals for the cells. It is this very approach that is determining the dynamic development of the elaboration of systems for the delivery of cells and scaffolds based on biomaterials in combination with stem cells [15].

In 2019, in the book “Definitions of Biomaterials for the Twenty-first Century”, a scaffold was defined as “a biomaterial structure that serves as a substrate and a guide for tissue repair and regeneration” [16]. This concept includes a rather extensive concept of scaffolds includes more specific formulations, for example, the concept of scaffolds “as products of tissue engineering, which are three-dimensional structures based on natural and/or synthetic materials and having certain characteristics of size, shape, mechanical, physical and chemical, and biological properties necessary for tissue repair” [17]. This new definition of a scaffold includes the “niche” concept. If a scaffold does not form an appropriate “niche” for the cells encapsulated in it in vitro or recruited from surrounding tissues after implantation in vivo, then it will not be able to provide for the anticipated tissue repair and regeneration. A scaffold, as an artificial extracellular matrix, can influence stem cell choices, such as rest or self-renewal, migration, proliferation, phenotype maintenance, differentiation, or apoptosis [18,19]. Therefore, the concept of a scaffold, as an artificial niche, involves not just maintaining cell viability, but the development of various cellular events depending on the signals received from the scaffold, both mechanical and biochemical [20,21]. At the same time, cells in vivo can influence their local niche by maintaining its homeostasis and transforming their niche by changing its microenvironment and regulating cellular processes using feedback mechanisms [22,23]. Cells can transform their microenvironment in a way that can largely determine their regenerative effect [24]. To that end, it is important that the scaffold used should enable the cells to transform their microenvironment, for example, by the secretion of various proteins, or by causing changes in the structure of the scaffold, and its properties. We can assume that only with the above-mentioned properties, can scaffolds be considered an artificially created specific tissue-like microenvironment for the cells or an artificial biomimetic cell niche [25,26]. Understanding scaffolds as an artificial niche may be key to developing tissue-engineering products with high regenerative potential.

In our work, we present a hydrogel biopolymer scaffold, formed from a cryoprecipitate of blood plasma and collagen under conditions of enzymatic hydrolysis, including encapsulated ASCs. We also review the biopolymer hydrogel scaffold as an artificial niche for mesenchymal stem cells. The scaffold has been shown to provide for the three-dimensional growth of the ASCs, allowing them to maintain high viability and proliferative and secretory activity. During the cultivation of the scaffold, changes in the structure of the scaffold and features of its biodegradation have been observed. The revealed changes are similar to the natural processes of "dynamic reciprocity", which is characteristic of a cell niche.

## 2. Materials and Methods

The study protocol was approved by the Local ethical committee of the FSBEI HE PRMU MOH (Nizhny Novgorod, Russia) and confirmed by its Academic Board (State assignment No. АААА-А18-118052190095-5, approved by the local ethics committee on 27 June 2017, protocol No. 8). Each person included in the study provided voluntary informed consent for the specified, following manipulations.

All manipulations with blood and its derivatives and the extraction and cultivation of cells regarding the formation and cultivation of hydrogel scaffolds were carried out under sterile laminar conditions (class A) in the biotechnologies laboratory of the FSBEI HE PRMU MOH (Nizhny Novgorod, Russia). At all stages of the study, the sterility of the materials the media used was controlled. Sterility control was carried out by examining samples for the presence of mycoplasmas, viruses, bacteria, and fungal microflora using both the PCR method and bacterial studies.

### 2.1. Blood Plasma

Blood plasma was obtained from the GBUZ NO blood center (Nizhny Novgorod, Russia) "Nizhny Novgorod Regional Blood Center named after N.Ya. Klimova". All donors were examined by specialists and examined for vector-borne infections. The resulting plasma was stored at a temperature of −40 °C. Blood plasma was defrosted at 2 °C and centrifuged at 4 °C for 15 min at 1500 rpm to separate the cryoprecipitate. Eighty-five percent of the supernatant was removed from the initial volume of frozen blood plasma. The isolated cryoprecipitate was then maintained at 37 °C until it was completely dissolved. The cryoprecipitate was standardized by the amount of fibrinogen to a final concentration of 6 g/L [27]. While carrying out the experiment we used a pool of cryoprecipitate obtained from eight donors.

### 2.2. Cell Cultures

Fat tissue obtained during cosmetic operations (three women; aged from 20 to 34 years) in the Department of Reconstructive and Plastic Surgery at the University Hospital of the FSBEI HE PRMU MOH was used as the initial material from which to obtain mesenchymal stem cells.

Adipose stem cells (ASCs) were isolated from this adipose tissue obtained while conducting plastic surgery operations. The cells were extracted using heat enzymatic processing with collagenase (Sigma-Aldrich, Darmstadt, Germany) and cultivated in complete growth medium (20% fetal bovine serum (FBS), α-MEM, glutamine, antibiotics (LLC PanEco, Moscow, Russia)) at 37 °C in a humid atmosphere with 5% CO_2_. Cultures from the third passage were used for the experiments. ASCs with confirmed differentiation potential were used in the research. The differentiation potential of the cells was evaluated on cultures of the third passage [27]. Before starting the experiment, we defined the cell phenotypes. The immunophenotype of the cells was characteristic of ASCs: the cells expressed CD90+, CD105+, CD 73+, and CD 44+ and did not express CD 45−, CD 14−, CD 34−, HLA DR-, and this corresponded to the criteria defined by the International Society for Cellular Therapy for Mesenchymal Cells [28].

### 2.3. Hydrogel Scaffold Formation

For the formation of scaffolds, blood plasma cryoprecipitate obtained from healthy donors was used [27,29]. The plasma cryoprecipitate was PEGylated using PEG-NHS (Sigma-Aldrich, Darmstadt, Germany). Then a 2% collagen solution was added (PH = 7.4). In our research, type I collagen isolated from cod skins was used [30,31]. The resulting composite was injected with a cell suspension in phosphate buffer. The concentration of cells per 1 mL of the composite was 1.2 × 10^5^. The formation of scaffolds took place under the conditions of an enzymatic hydrolysis reaction. The composite was injected with a thrombin-calcium mixture: 80 IU/mL of human thrombin (NPO RENAM, Moscow, Russia) in 1% CaCL_2_ solution. Scaffolds were formed within 20 min at a temperature of 22 to 25 °C. The scaffolds were then transferred to a plastic Petri dish. The scaffolds were cultured in complete growth medium. The cultivation was carried out in a CO_2_ incubator at 37 °C, with a humidified atmosphere, and 5% CO_2_ content.

**Figure 1:**
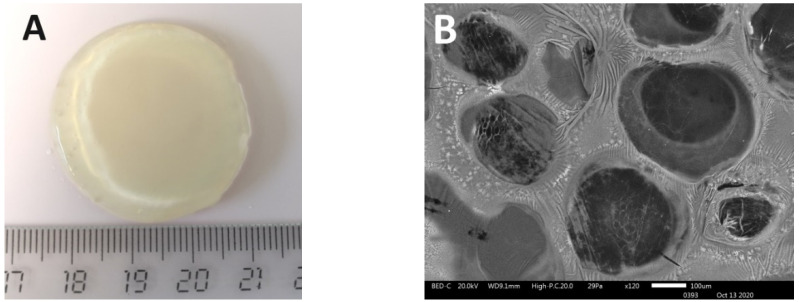
(**A**) The appearance of the scaffolds. (**B**) Internal architecture of scaffolds. Cross-section samples of dehydrated scaffolds were visualized using a scanning electronic microscope (JSM-IT300; JEOL Ltd., Japan), and dehydration of the samples was performed in the chamber of the JSM-IT300 under low vacuum.

The resulting scaffolds were dimensionally stable and transparent (Figure 1A). The internal structure of the scaffolds was characterized by their heterogeneous porosity (Figure 1B).

### 2.4. Scanning Electronic Microscopy

The investigation of the scaffold samples (*n* = 3) was carried out with a JSM-IT300 (JEOL Ltd., Tokyo, Japan) scanning electron microscope. Samples of dehydrated scaffolds were visualized and the dehydration of the samples was performed in the chamber of the JSM-IT300 under a low vacuum.

### 2.5. Comparative Characteristics of the Porosity of the Structure of Scaffolds

To carry out a comparative characterization of the porous scaffold structure (*n* = 3), microphotographs obtained by electron transmission microscopy (14,000×) were used. The scaffolds were cultured for 10 days under standard conditions. The control period (1, 3, 6, and 10 days) fragments, which were prepared for transmission microscopy, were removed from the scaffolds. The preparation of these samples and their study were carried out according to standard methods. Samples were fixed in a 2.5% solution of glutaraldehyde in phosphate buffer (pH = 7.4) and in a 1% solution of osmium tetroxide, before being dehydrated in alcohols of ascending concentration (from 50 to 100%) and acetone (100%). Then they were kept in a mixture of 50% embedding medium and 50% acetone, followed by further embedding in a mixture of Epona with Araldite. After polymerization, we obtained ultrathin slices 75 to 80 nm thick on a UC7 (Leica Microsystems, Wetzlar, Germany) ultratome and observed them with a Morgagni 268D transmission electron microscope (FEI, Hillsboro, OR, USA). Microphotographs (*n* = 20 for each sample point) were processed using ImageJ software (version 1.50i, Wisconsin, National Institutes of Health, Bethesda, MA, USA). When analyzing microphotographs, the threshold binarization procedure was used to distinguish the area of interest (the biopolymer part of the scaffold) and the background image (pore lumen). Following scanning of the entire image field, taken as 100%, the percentages of the biopolymer part of the scaffold and the pore lumen in the structure of the scaffold were calculated.

### 2.6. Fluorescence Microscopy

To visualize the cells, confirm their viability, and to characterize the cytoskeleton of the cells cultured within the structure of scaffolds (*n* = 5), we used fluorescence microscopy carried out on a Cytation 5 (BioTek, Winooski, VE, USA) multifunctional imager. For the visualization of viable cells, Calcein AM (catalog No. 564061, BD, Franklin Lakes, NJ, USA) was used (excitation wavelength of 477 nm and emission wavelength of 525 nm). Staining was carried out in accordance with the manufacturer’s protocol. Invitrogen ™ Alexa Fluor™ 594 Phalloidin (catalog No. 12381, Thermo Fisher, Waltham, MA, USA; excitation wavelength 586 nm and emission wavelength 647 nm) was used to visualize the cytoskeleton. When carrying out a quantitative analysis of the cells, fluorochrome staining of the cell nuclei was used: for the total number of cells—Hoechst 3334 (catalog No. 561908, BD, Franklin Lakes, NJ, USA; excitation wavelength of 377 nm and emission wavelength of 477 nm); number of dead cells—NucGreenTM Dead 488 (catalog No. R37109, Invitrogen by Thermo Fisher Scientific, Waltham, MA, USA; excitation wavelength of 477 nm and emission wavelength of 525 nm).

### 2.7. Quantitative Analysis of Cells in Scaffolds

To characterize the numbers of cells within the structure of the hydrogel scaffolds and to evaluate their proliferative activity during cultivation, fragments with an area of 0.64 cm^2^ were separated from the test samples taken after the relevant incubation period using a template. The number of cells was determined by counting the nuclei in the selected fragment [32]. We analyzed micrographs taken from several fields of view at arbitrary regions within the thickness of the samples. Fluorescence microscopy was performed using the Z-stack function. The following objects were recorded: nuclei of all cells (staining with Hoechst 3334; magnification: 4× objective, 10× eyepiece; depth of the analyzed layer along the Z-axis 530 µM), as well as the nuclei of dead cells (staining with NucGreenTM Dead 488; magnification: 10× objective, 10× eyepiece; depth of the analyzed layer along the Z-axis 300 µM). Quantitative analysis was carried out using cross-linked Z-stack micrographs. The number of cell nuclei was counted and subsequently converted to the number of cells per 1 mm^3^.

### 2.8. Quantification of VEGF in the Growth Medium

To determine the secretory activity of the ASCs cultured in the scaffolds, their ability to produce the VEGF-A factor, one of the vascular endothelial growth factor (VEGF) family, was studied. Scaffolds (*n* = 6) were cultured under standard conditions for seven days without changing the growth medium. Samples of the research medium were taken, and a quantitative analysis of the cells was performed at three to seven days from the moment of scaffold formation and the beginning of its cultivation (day zero). The growth medium samples taken prior to the start of research were subject to obligatory control. To quantify the content of VEGF-A (“eBioscience”, Thermo Fisher, Waltham, MA, USA) reagent kits were used. The optical density value was recorded on a Tecan analyzer (Tecan Group Ltd., Männedorf, Switzerland) using the Magellan program.

### 2.9. Scaffold Biodegradation Study

The biodegradation of scaffolds was studied in two types of solutions: of phosphate buffer solution—passive biodegradation and 0.25% trypsin solution in Versen’s solution (LLC PanEco, Moscow, Russia)—biodegradation under the action of a hydrolytic enzyme. Samples with a diameter of 8 mm were isolated from scaffolds with the use of a template and placed in 24-well plates. For the study, scaffold samples were used that had previously been cultured with the ASCs under standard conditions for different periods: 24 h of cultivation (*n* = 14) and six days (*n* = 14). One milliliter of PBS or 1 mL of trypsin solution was added to the wells with the scaffold samples. The sample plates were placed in a CO_2_ incubator and incubated at 37 °C, with a humidified atmosphere and 5% CO_2_. At sample times of 2, 4, 6, 24, 48, 120, 480, 720, 840, and 1008 h, a sample of 100 μL in an Eppendorf tube was taken from each well and frozen at a temperature of −80 °C for subsequent examination. After sampling, the volume of liquid in each well was replenished with 100 μL of the appropriate solution. The degree of biodegradation was estimated by the total amount of free protein identified in the samples. For this, the samples were defrosted at room temperature for 24 h, then the protein concentration in the samples was assessed on an IRF-456 refractometer (KARAT MT, Moscow, Russia) in accordance with a calibration graph. This calibration graph had been generated using samples of human serum albumin (Baxter AG, Vienna, Austria) of known concentration [33].

### 2.10. Statistical Analysis

The results of the investigations were processed using non-parametric statistics methods and a Wilcoxon’s paired-comparison test and Spearman correlation analysis—RS (STATISTICA (data analysis software system) version 6.0; Dell Technologies Inc., Round Rock, TX, USA).

## 3. Results

**Figure 2:**
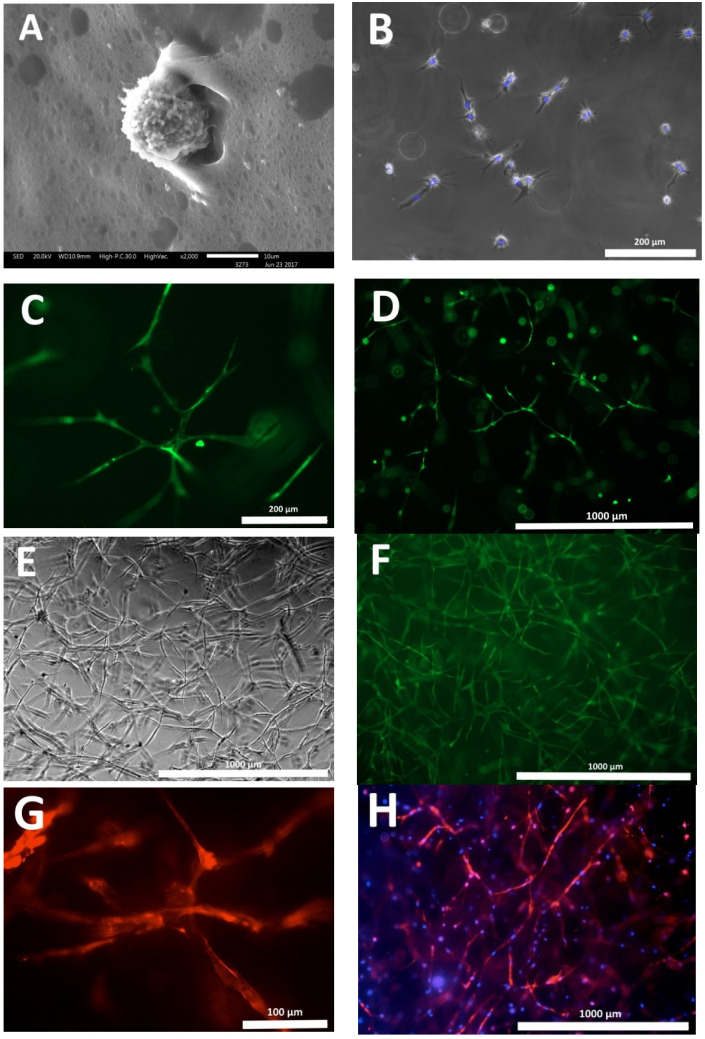
Cultivation of scaffolds with encapsulated ASCs. (**A**) ASCs adhered within the scaffold structure, after 24 h of cultivation, scanning electronic microscopy; (**B**) ASCs in the scaffold structure after 48 h of cultivation, here the cells have formed multiple processes. Phase-contrast combined with fluorescence microscopy, cell nuclei stained with Hoechst 3334—blue, (**C**,**D**) ASCs in the scaffold structure—third day of cultivation, cells exhibit three-dimensional growth and form intercellular junctions, (**E**,**F**) ASCs in the scaffold structure—sixth day of cultivation, cellular network, (**G**) cells in the scaffold structure—eighth day of cultivation, the well-developed cell cytoskeleton is clearly visualized (**H**) as a cell network formed by the ASCs with a developed cytoskeleton in the scaffold structure—eighth day of cultivation. (**C**,**D**,**F**) Calcein AM staining confirms cell viability (fluorescence microscopy), (**G**,**H**) staining: red—cell cytoskeleton, Invitrogen™ Alexa Fluor™ 594 Phalloidin; blue—cell nuclei, Hoechst 3334 (fluorescence microscopy).

Hydrogel scaffolds formed according to the above method with encapsulated ASCs were cultured in vitro (37 °C, in a humidified atmosphere and 5% CO_2_) with a change in growth medium twice a week. While in scaffold cultivation, the ASCs exhibited matrix–cell adhesion (Figure 2A,B), but ASCs are surface dependent cells, and in the free state (detached) they are spherical without processes. Only after adhesion to the surface do the ASCs form outgrowths. In Figure 2B, there are clearly visible cells with multiple processes, indicating their adhesion to the structural elements of the scaffold. During subsequent cultivation, the ASCs showed three-dimensional growth and formed intercellular contacts (Figure 2C,D). On the sixth day, a developed cellular network could be observed in the scaffolds (Figure 2E,F,H). Also, staining of the cells with Calcein AM demonstrated the viability of the ASCs cultured in the scaffolds (Figure 2C,D,F). The percentage of viable cells in scaffolds during cultivation was more than 95% (Table 1). During the cultivation of the scaffolds as described, the ASCs had developed an organized cytoskeleton that was easily visualized when stained with a specific fluorochrome (Figure 2G,H).

**Figure 3:**
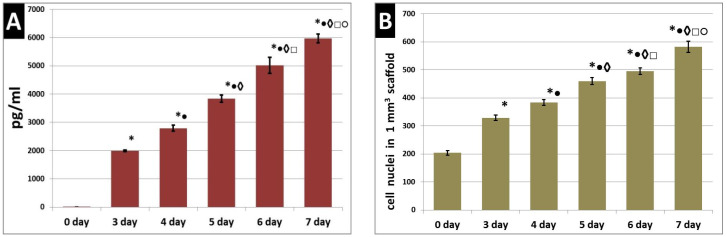
Functional and proliferative activity of ASCs cultured in scaffolds. (**A**) Secretion of VEGF-A by ASCs cultured in scaffolds; (**B**) number of cell nuclei in the per 1 mm^3^ scaffold. Note: *—*p* ˂ 0.05 comparison with day zero, •—comparison with day three, ◊—comparison with day four, □—comparison with day five, ○—comparison with day six, Wilcoxon test.

During their cultivation in the scaffolds, the functional activity of the ASCs was evaluated by their ability to secrete VEGF-A. It was found that ASCs cultured in scaffolds for seven days actively secreted VEGF-A, this being detected in the culture medium from the third to the seventh day (Figure 3A). In the same series of scaffold samples (*n* = 3), cultivated in parallel under the same conditions, the number of cells per 1 mm^3^ of the scaffold was analyzed (using *n* = 11 analyzed fields of view from each scaffold for each sample period). It was demonstrated that a statistically significant increase in the number of cell nuclei per 1 mm^3^ of the scaffold could be observed with an increase in cultivation time (Figure 3B). This indicated the maintenance of pronounced proliferative activity of the ASCs cultured in the scaffolds. An analysis of the correlation of the number of cells per 1 mm^3^ of scaffold and the VEGF-A content in the culture medium of each confirmed the presence of a positive correlation between the analyzed parameters (RS = 0.844; *p* = 0.000000).

**Figure 4:**
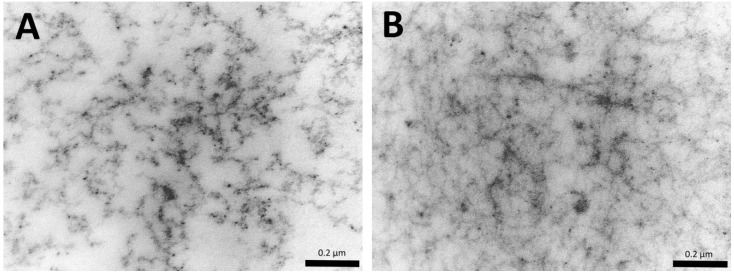
Change in the structure of scaffolds: (**A**)—first day of cultivation, (**B**)—10th day of cultivation (transmission electron microscopy).

**Figure 5:**
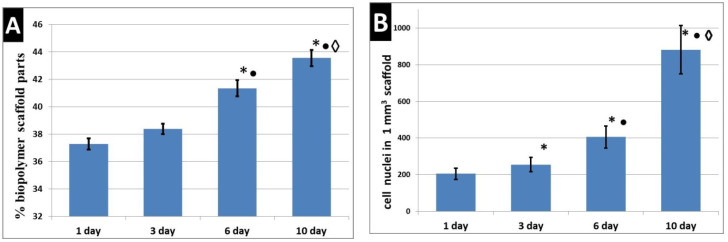
Change in the structure of scaffolds and the number of cells in the scaffolds during cultivation. (**A**) Changes in the structural characteristics of scaffolds. (**B**) Changing the number of cells in the scaffolds. Note: *—*p* ˂ 0.05 comparison with day one, •—comparison with day three, ◊—comparison with day six, Wilcoxon test.

The change in the structural characteristics of the cellular scaffolds during their cultivation was assessed. It was found that, over time, there was a compaction of the scaffold structure (Figure 4). By day six, the percentage of the biopolymer portion of the scaffolds had increased by 9% compared to day one (Figure 5A). With the cultivation of the scaffolds for a further day, even more pronounced changes were observed. On the 10th day, the percentage of the biopolymer part of the scaffold was 15% higher than on the first day. In the same series of scaffolds (*n* = 3), cultivated in parallel under the same conditions, we analyzed the number of cells per 1 mm^3^ of the scaffold (using *n* = 15 analyzed visual fields from each scaffold sample from each sample time). It was shown that there was an increase in the number of cell nuclei per 1 mm^3^ of scaffold over the cultivation time (Figure 5B). Correlation analysis of the percentage of the scaffold biopolymer part and the number of cells per 1 mm^3^ confirmed the presence of a positive correlation between the analyzed parameters (RS = 0.776; *p* = 0.000000).

**Figure 6:**
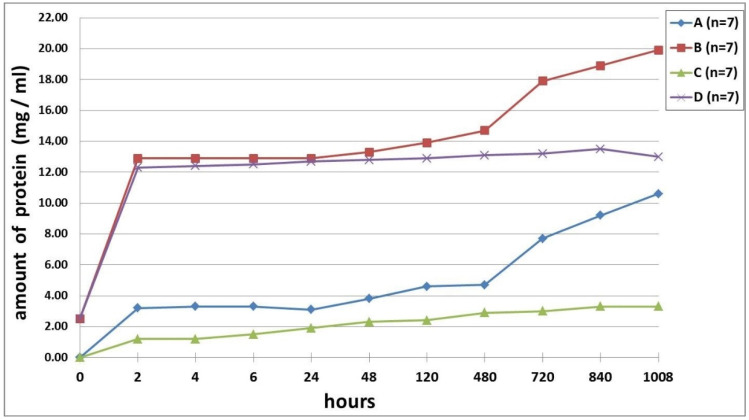
Biodegradation of hydrogel scaffolds in vitro. (**A**,**C**) Passive biodegradation of scaffolds in a PBS solution. (**B**,**D**) Biodegradation of scaffolds by the action of a hydrolytic enzyme—trypsin. (**A**,**B**) Biodegradation of scaffolds administered into the experiment after 24 h of cultivation. (**C**,**D**) Biodegradation of scaffolds, administered into the experiment after cultivation for six days. Note: The percentage of mistake of the determination was 0.02%.

A study of the passive (in PBS solution) and enzymatic hydrolytic biodegradation (in trypsin solution) of the hydrogel scaffolds was conducted at various stages of cultivation. It was demonstrated that the scaffolds used in the experiment, under the action of the enzyme degraded much more actively than in PBS solution at each stage of cultivation (Figure 6). However, depending on the stage of cultivation, certain differences in the biodegradation of the scaffolds were revealed. Thus, after 24 h of cultivation, the scaffolds were more exposed to passive degradation (Figure 6, series A—passive degradation in PBS of the 24 h scaffold cultures). Even after 2 h, the protein concentration determined in the medium in which the scaffolds were incubated in series A was 2.67 times higher than in series C (passive degradation in PBS of the six-day scaffold cultures). The dynamics indicated a gradual increase in the amount of protein released during the biodegradation of the series A scaffolds over 480 h, with some statistically insignificant fluctuations, for example, at 24 h. At later times (720 to 1008 h), a sharp increase in the amount of protein in the medium was observed, indicating a more marked decomposition of the scaffolds. So, in the period from 480 h to the end of the experiment (1008 h), the amount of protein increased 2.26-fold. Compared with the beginning of the experiment (2 h), the amount of protein in the medium by the end of the experiment had increased more than three-fold. A different pattern was observed with the passive biodegradation of the series C scaffolds. So, starting from two h until the end of the experiment, the protein concentration gradually increased and reached a plateau after 840 h. Compared with the beginning of the experiment (two hours), the amount of protein in the medium by the end of the experiment had increased 2.75-fold. At the final point, the protein concentration in the medium from the series C scaffolds was 68.86% lower than that in series A.

When assessing the biodegradation of the corresponding scaffolds under conditions of enzymatic hydrolysis, it was revealed that scaffolds with preliminary cultivation for 24 h (series B) degraded more rapidly than scaffolds with preliminary cultivation for six days (series D) (Figure 6). The key points in biodegradation of the scaffolds under the action of the enzyme were similar to those that we observed in the passive biodegradation of the scaffolds. So, after two hours, a sharp increase in the protein concentration was recorded compared with the zero point (trypsin solution). The protein concentration, at two hours, in the medium from the series B scaffolds was 12.9 mg/mL, and that of series D was 12.3 mg/mL, with there being no statistically significant differences between the series. In group B, from 2 to 120 h inclusive, an increase in the amount of protein in the medium, with small fluctuations, was observed. In samples of the medium taken 480 h after the start of the experiment, there was an increase in the amount of protein (5.8%) compared with the samples taken after 120 h. After this, and until the end of the experiment, the protein concentration increased sharply. By the end of the experiment (1008 h), the amount of protein in series B (24-hour pre-cultivation) had increased by 43% compared to 120 h and by 54% compared to 2 h. The series D (six-day pre-cultivation) scaffolds showed different biodegradation dynamics in the enzyme solution from series B. The amount of protein in the samples gradually increased over time (up to 840 h inclusive), and, starting from six hours, these changes were statistically significant. At the end of the experiment (1008 h), a decrease in protein concentration was observed. This outcome appears to be a result of the methodology that involved replenishing the amount of liquid in the well with the incubated sample with the same volume of liquid as had been taken in the sample (100 μL). Thus, if protein release had ceased or was extremely small, a dilution effect would be observed. Based on the results, we can conclude that the biodegradation of series D scaffolds was completed by 35 days (840 h). The amount of protein in the period from 2 to 840 h increased by 9.7%. It should be noted that the maximum protein concentration in series B (1008 h) was 47% higher than that in series D (840 h).

In the study of the correlation between the series A and B scaffolds, the presence of a very high positive correlation RS = 0.917 (*p* = 0.000000) was confirmed. The correlation between series C and D was 16% lower and amounted to RS = 0.778 (*p* = 0.000000). The presence of a positive correlation was also confirmed between series B and D—RS = 0.789 (*p* = 0.000000) and between series A and C—RS = 0.815 (*p* = 0.000000).

**Figure 7:**
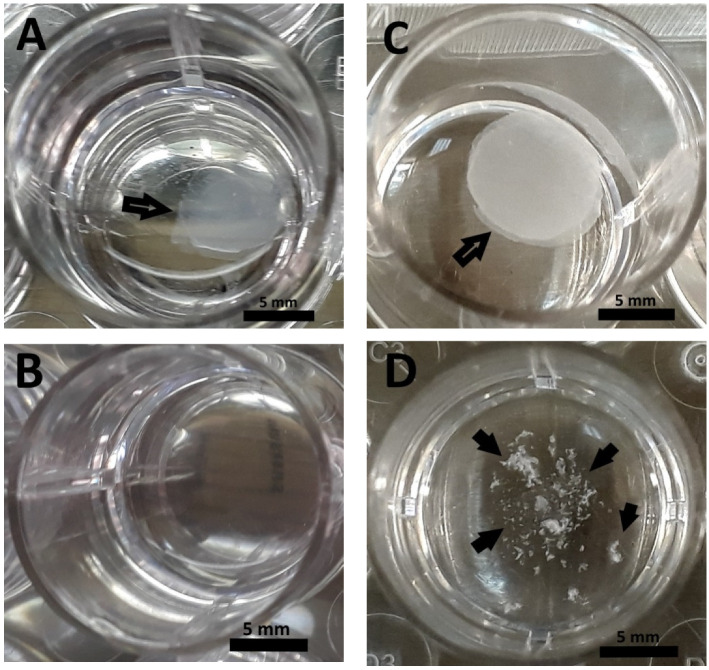
Appearance of scaffold samples after 1008 h of biodegradation in vitro: (**A**)—series A, (**B**)—series B, (**C**)—series **C**, (**D**)—series D. Arrows indicate scaffolds and their fragments.

The data obtained corresponded to a visual assessment of the state of the scaffolds. So, the samples of series A and series C in PBS solution at the end of the experiment (1008 h) still clearly showed the scaffold retaining its initial oval shape (Figure 7A,C). The scaffolds in the series B samples in the trypsin solution could not be visualized at the end of the experiment, and no fragments could be observed (Figure 7B). Residual fragments of scaffolds were observed in the wells with the series D samples and were clearly visible by visual inspection of the wells with samples (Figure 7D).

## 4. Discussion

Stem cells normally operate in a special biologically active extracellular environment that provides regulation of their cellular processes using a variety of signals. Our paper presents a biopolymer hydrogel scaffold formed on the basis of a cryoprecipitate of blood plasma and collagen—biologically active components of natural origin. The main scaffold structure-forming proteins are fibrinogen/fibrin and collagen [27]. These are adhesive peptides that can activate integrin receptors on the surfaces of cells. Integrins are known to be heterodimeric transmembrane receptors capable of binding to extracellular matrix proteins or soluble extracellular ligands and integrating the information obtained from them into a network of signal chains that coordinate cell behavior [34,35]. For example, α2β1 can bind to collagen, and αvβ3 can bind to several ligands, such as fibronectin, vitronectin, and fibrinogen. There are RGD sequences in fibrinogen/fibrin and collagen that interact with the integrin receptors of cells, and thus regulate many cellular processes (viability, adhesion, migration, and proliferation) [36,37,38,39]. Collagen, in its turn, has integrin-recognizing sequences represented by high-affinity GxOGER triple-helical sequences that interact with the cells through β1-containing integrins (α1β1, α2β1, α10β1, and α11β1) [40,41,42]. It should be noted that the binding of integrins to ligated sequences of collagen requires the presence of divalent cations [43]. The presence of blood plasma cations and exogenous calcium used in the formation of the scaffold can promote an increase in the affinity of RGD and the GxOGER collagen ligand with the integrin cell receptors.

It is known that blood plasma, which formed the basis of the presented scaffold composite, contains amino acids necessary for the growth and proliferation of cells, [44] together with proteins, such as fibrinogen and fibronectin—one of the key proteins of the intercellular matrix [45]. According to published data, the concentration of fibrinogen, fibronectin, factor XIII, fibrinolysis inhibitors, and circulating cell adhesion molecules in cryoprecipitate of blood plasma is much higher than in blood plasma [46,47]. In addition to their ability to interact directly with stem cells, extracellular matrices (ECMs) are also able to regulate stem cell activity by presenting “noncanonical” growth factors [10]. Some ECM components can actively bind growth factors, regulating their local availability, and establishing a biochemical gradient [48]. In this case, the ECM can function as a reserve of growth factors. Several studies have demonstrated the ability of the fibrin matrix to act as a natural carrier of contained cellular protein factors, including those found in blood plasma: fibroblast growth factor (FGF), platelet-derived growth factor (PDGF), transforming growth factor-beta (TGF-β), and thrombospondin-1 (TSP1) [49,50,51,52,53]. There is evidence that the fibrin matrix can balance the relative concentrations and affect the controlled mass-dependent release of such factors [54]. The ability of fibrinogen/fibrin to bind and release protein factors can provide long-term regulation of cellular responses within a scaffold. Thus, the development of cellular events (the manifestation of adhesion, maintenance of viability, pronounced proliferative activity of ASCs, etc.) in the presented scaffold can be largely determined by the signals received by the cells from the biologically active scaffold, and then be maintained due to the presence of a complex of protein factors initially present in the cryoprecipitate of blood plasma, and be released over a prolonged period by the biopolymer matrix.

Several studies have demonstrated the direct effect of the microstructure of scaffolds on cell adhesion, migration, and proliferation [55,56]. We have previously demonstrated that the internal structure of the presented scaffolds is formed by sufficiently thick interconnected fibers and has a developed system of interconnected heterogeneous pores [27]. Thus, the three-dimensional structure of such scaffolds promotes the maintaining of the viability and proliferative activity of the ASCs, while providing mechanical support and suitable conditions for the placement and interaction of the cells. The hydrophilic nature of scaffolds, being hydrogels, makes it possible for them to retain a large amount of liquid. This property facilitates the exchange of nutrients and the waste products of the cells, therefore also helping to maintain cellular events.

During cultivation, the ASCs encapsulated in the scaffolds demonstrated three-dimensional growth with the formation of multiple processes and intercellular connections. This is consistent with other studies on the behavior of mesenchymal stem cells encapsulated in three-dimensional hydrogel scaffolds [57,58]. One of the key characteristics of MSCs is the state of the cytoskeleton. The cytoskeleton acts not only as a scaffold but also plays an important role in the transport of organelles, cell division, migratory activity, and signal transmission [59,60]. Cytoskeletal systems are the main mechanosensors (focal adhesions) and mechanotransmitters (actin fibers and microtubules) of a unique mechanism of mechanotransduction. Mechanical transduction involves a cascade of intracellular molecular processes by which physical signals from the ECM or neighboring cells are converted into biological responses. Mechanotransduction allows cells to adapt promptly to dynamic changes in their microenvironment [61,62]. For example, mechanotransduction is crucial for modulating the stem cell phenotype under conditions of changing the geometric conformation of the ECM, which is directly associated with a change in mechanical stimuli [63,64]. We have previously demonstrated that after the cultivation of ASCs within the structure of the presented scaffold, a significant (from 60 to 80%) decrease in the proportion of cells expressing CD90 can be observed, without changing the proportion of CD73+, CD105+, CD 44+, or other cells [65]. After culturing ASCs isolated from the scaffold for 96 h on plastic, the proportion of cells expressing CD90 was restored to 95 to 96%. The latter corresponded to the phenotype of the cell culture before encapsulation in the scaffold. Thus, three-dimensional cultivation in a scaffold had changed the phenotype of the ASCs, this apparently is associated with the response of the cells to signal changes during the transition from a 2D culture to a scaffold and back to the culture. Several studies have demonstrated that the cytoskeletal system of actin filaments, which is the main component of the cytoskeleton responsible for cell stiffness, undergoes extensive remodeling in the process of differentiation [66,67]. In their normal state, undifferentiated MSCs have elongated fibers that run mostly parallel to the long axis of the cell. Cells cultured in scaffolds had a developed cytoskeleton corresponding to their morphology, specifically attributed to MSCs, which indicated the maintenance of the ASCs in an undifferentiated state.

According to published data, cell adhesion and intercellular junctions are necessary conditions for proper metabolism and protein synthesis [68]. It has been demonstrated that ASCs in scaffolds adhere and form intercellular junctions resulting in a developed cell network. When cultivating ASCs in scaffolds, we confirmed that the cells synthesized actively and secreted VEGF-A. VEGF-A is a key pro-angiogenic factor [69], that, along with other growth factors, is synthesized by MSCs both in vitro and in vivo [70,71]. Cells cultured in scaffolds proliferated actively, and the level of VEGF-A, determined in the culture medium correlated with the number of cells in the scaffolds. Thus, the ASCs in these scaffolds not only maintained viability and specific morphological characteristics but also changed their microenvironment, proliferating, and secreting VEGF-A. Moreover, it was demonstrated that the cells actively changed the structural characteristics of the scaffold, leading to densification of its structure. At the same time, the cells continued to proliferate actively. The presence of a positive correlation between the percentage of the biopolymer part of the scaffold and the number of cells was demonstrated. Thus, not only did the scaffold affect the cells, but we also observed feedback—the cells affected the scaffold. Under the influence of the cells, changes in the biodegradation properties of the scaffolds were observed. It was demonstrated that scaffolds with cells cultured previously for only 24 h degraded more rapidly than scaffolds that had been allowed to be remodeled by cells in culture for six days. A decrease in the correlation coefficient determined between passive and enzymatic degradation in scaffolds cultivated for six days compared to scaffolds cultivated for only 24 h indicated a change in the nature of the biodegradation process. The correlation factors determined between scaffold groups cultured for 24 h and for six days in phosphate buffer (series A and C) and trypsin (series B and D) demonstrated a fairly strong positive relationship. At the same time, the factors were significantly lower than one, which also confirmed the presence of differences in the biodegradation properties of the scaffolds of these groups. The latter could be due both to a change in the structural characteristics of the scaffold and a change in its composition. It is known that MSCs secrete not only VEGF-A but also a range of other proteins. This feature determines their paracrine regenerative potential and allows remodeling of the ECM, maintaining homeostasis, or providing for the restoration of damaged tissues [72]. Thus, natural ECMs are constantly being rounded through a process called “dynamic reciprocity” [24]. The presence of “dynamic reciprocity” between ASCs and an artificial ECM—the scaffold, allows us to consider the presented scaffold as an appropriate artificial biomimetic niche for MSCs.

## 5. Conclusions

A hydrogel scaffold based on a cryoprecipitate of blood plasma and collagen with encapsulated ASCs has been presented. In the process of scaffold cultivation with ASCs, the cells showed matrix–cell adhesion, three-dimensional growth with the formation of a cell network, and had a developed cytoskeleton. It can be assumed that successful cellular events were provided for by the biologically active components of the scaffold and its structural characteristics. It was found that ASCs secreted VEGF-A dynamically and changed the structural characteristics of the scaffold. Long-term cultivation of scaffolds with encapsulated ASCs led to a change in the biodegradation properties of the scaffolds. Summarizing the results obtained, we can conclude that the processes that occurred during the cultivation of the scaffold with the ASCs were similar to the natural processes of “dynamic reciprocity”. This enables us to consider the presented scaffold as an appropriate artificial niche for such cells. We hope that the properties of the scaffolds presented in this research, which can match a natural niche for ASCs, will serve as the basis for developing scaffolds with good regenerative potential in the future. The integrated approach presented in this research can also be used to characterize other scaffolds from the perspective of their providing of effective artificial types of cell niche.

## Figures and Tables

**Table 1:** ASC viability in scaffolds.

Cultivation	Total Number of Cell Nuclei per 1 mm^3^ Scaffold	Number of Nuclei of Dead Cells per 1 mm^3^ Scaffold	% of Nuclei of Dead Cells
72 h	292.07 ± 15.51	2.33 ± 0.31	0.79
144 h	636.79 ± 33.85	3.60 ± 0.42	0.56

Note: The study was conducted on nine scaffold samples. Five fields of view were analyzed from each scaffold for each sample period.

## References

[1] Krishna L., Dhamodaran K., Jayadev C., Chatterjee K., Shetty R., Khora S.S., Das D. (2016). Nanostructured scaffold as a determinant of stem cell fate. Stem Cell Res. Therapy.

[2] Drela K., Stanaszek L., Nowakowski A., Kuczynska Z., Lukomska B. (2019). Experimental Strategies of Mesenchymal Stem Cell Propagation: Adverse Events and Potential Risk of Functional Changes. Stem Cells Int..

[3] Choi J.R., Yong K.W., Nam H.Y. (2019). Current status and perspectives of human mesenchymal stem cell therapy. Stem Cells Int..

[4] Fitzsimmons R.E.B., Mazurek M.S., Soos A., Simmon C.A. (2018). Mesenchymal stromal/stem cells in regenerative medicine and tissue engineering. Stem Cells Int..

[5] Sávio-Silva C., Soinski-Sousa P.E., Balby-Rocha M.T.A., Lira Á.D.O., Rangel É.B. (2020). Mesenchymal stem cell therapy in acute kidney injury (AKI): Review and perspectives. Revista da Associacao Medica Brasileira.

[6] Regmi S., Pathak S., Kim J.O., Yong C.S., Jeong J.-H. (2019). Mesenchymal stem cell therapy for the treatment of inflammatory diseases: Challenges, opportunities, and future perspectives. Eur. J. Cell Biol..

[7] Pittenger M.F., Discher D.E., Péault B.M., Phinney D.G., Hare J.M., Caplan A.I. (2019). Mesenchymal stem cell perspective: Cell biology to clinical progress. NPJ Regen. Med..

[8] ClinicalTrials.gov. https://www.clinicaltrials.gov/ct2/home.

[9] Gomez-Salazar M., Gonzalez-Galofre Z.N., Casamitjana J., Crisan M., James A.W., Péault B. (2020). Five Decades Later, Are Mesenchymal Stem Cells Still Relevant?. Front. Bioeng. Biotechnol..

[10] Taddei M.L., Giannoni E., Fiaschi T., Chiarugi P. (2012). Anoikis: An emerging hallmark in health and diseases. J. Pathol..

[11] Gattazzo F., Urciuolo A., Bonaldo P. (2014). Extracellular matrix: A dynamic microenvironment for stem cell niche. Biochim. Biophys. Acta Gen. Subj..

[12] Pennings S., Liu K.J., Qian H. (2018). The stem cell niche: Interactions between stem cells and their environment. Stem Cells Inter..

[13] Watt F.M., Huck W.T.S. (2013). Role of the extracellular matrix in regulating stem cell fate. Nat. Rev. Mol. Cell Biol..

[14] Pardo-Saganta A., Calvo I.A., Saez B., Prosper F. (2019). Role of the Extracellular Matrix in Stem Cell Maintenance. Curr. Stem Cell Rep..

[15] Zhao L., Hu C., Zhang P., Jiang H., Chen J. (2019). Preconditioning strategies for improving the survival rate and paracrine ability of mesenchymal stem cells in acute kidney injury. J. Cell. Mol. Med..

[16] Wagner W., Laurencin C., Lu H., Williams D., Simon C., Lu H., Best S., Williams D., Zhang X. (2019). Regenerative Medicine in Definitions of Biomaterials for the Twenty-First Century.

[17] Zhang X.D., Williams D.F. Definitions of Biomaterials for the Twenty-first Century. Proceedings of the Consensus Conference on Definitions of Biomaterials for the Twenty-First Century.

[18] Williams D.F. (2019). Challenges with the development of biomaterials for sustainable tissue engineering. Front. Bioeng. Biotechnol..

[19] Akhmanova M., Osidak E., Domogatsky S., Rodin S., Domogatskaya A. (2015). Physical, Spatial, and Molecular Aspects of Extracellular Matrix of in Vivo Niches and Artificial Scaffolds Relevant to Stem Cells Research. Stem Cells Int..

[20] Smith Q., Gerecht S. (2018). Extracellular Matrix Regulation of Stem Cell Fate. Curr. Stem Cell Rep..

[21] Nie Y., Zhang S., Liu N., Li Z. (2016). Extracellular Matrix Enhances Therapeutic Effects of Stem Cells in Regenerative Medicine. Compos. Funct. Extracell. Matrix Human Body.

[22] Sharma M., Ross C., Srivastava S. (2019). Ally to adversary: Mesenchymal stem cells and their transformation in leukaemia. Cancer Cell Inter. BioMed Central.

[23] Roberts K.J., Kershner A.M., Beachy P.A. (2017). The Stromal Niche for Epithelial Stem Cells: A Template for Regeneration and a Brake on Malignancy. Cancer Cell.

[24] Brown B.N., Badylak S.F. (2014). Extracellular matrix as an inductive scaffold for functional tissue reconstruction. Transl. Res..

[25] Aubert L., Dubus M., Rammal H., Bour C., Mongaret C., Boulagnon-Rombi C., Garnotel R., Schneider C., Rahouadj R., Laurent C. (2017). Collagen-based medical device as a stem cell carrier for regenerative medicine. Int. J. Mol. Sci..

[26] Hong K.H., Kim Y., Song S. (2019). Fine-Tunable and Injectable 3D Hydrogel for On-Demand Stem Cell Niche. Adv. Sci..

[27] Egorikhina M.N., Aleynik D.Y., Rubtsova Y.P., Levin G.Y., Charykova I.N., Semenycheva L.L., Bugrova M.L., Zakharychev E.A. (2019). Hydrogel scaffolds based on blood plasma cryoprecipitate and collagen derived from various sources: Structural, mechanical and biological characteristics. Bioact. Mater..

[28] Dominici M., Blanc K.L., Mueller I., Slaper-Cortenbach I., Marini F.C., Krause D.S., Deans R.J., Keating A., Prockop D.J., Horwitz E.M. (2006). Minimal criteria for defining multipotent mesenchymal stromal cells. The International Society for Cellular Therapy position statement. Cytotherapy.

[29] Egorikhina M.N., Levin G.Y., Charykova I.N., Alejnik D.Y., Sosnina L.N. (2018). Method for Creating a Bioresorbable Cellular Scaffold Based on Fibrin of Blood Plasma. Int. Cl. C12N 5/00 Bull.

[30] Semenycheva L.L., Astanina M.V., Kuznetsova J.L., Valetova N.B., Geras’kina E.V., Tarankova O.A. (2015). Method for Production of Acetic Dispersion of High Molecular Fish Collagen.

[31] Semenycheva L.L., Egorikhina M.N., Chasova V.O., Valetova N.B., Kuznetsova Y.L., Mitin A.V. (2020). Enzymatic hydrolysis of marine collagen and fibrinogen proteins in the presence of thrombin. Mar. Drugs.

[32] Egorikhina M.N., Charykova I.N., Alejnik D.Y. (2018). Method of Quantitative Analysis of Cellular Components of Scaffold. Int. Cl. G01N 33/52 Bull.

[33] Sharma V., Patel N., Kohli N., Ravindran N., Hook L., Mason C., García-Gareta E. (2016). Viscoelastic, physical, and bio-degradable properties of dermal scaffolds and related cell behaviour. Biomed. Mater..

[34] Liddington R.C., Ginsberg M.H. (2002). Integrin activation takes shape. J. Cell Biol..

[35] Prowse A.B.J., Chong F., Gray P.P., Munro T.P. (2011). Stem cell integrins: Implications for ex-vivo culture and cellular therapies. Stem Cell Res..

[36] Kaijzel E.L., Koolwijk P., van Erck M.G., van Hinsbergh V.W., de Maat M.P. (2006). Molecular weight fibrinogen variants determine angiogenesis rate in a fibrin matrix in vitro and in vivo. J. Thromb. Haemost..

[37] Laurens N., Engelse M.A., Jungerius C., Löwik C.W., van Hinsbergh V.W., Koolwijk P. (2009). Single and combined effects of alphavbeta3- and alpha5beta1-integrins on capillary tube formation in a human fibrinous matrix. Angiogenesis.

[38] Mork B.C., Vitebsky A., Hou M., Preising B. (2008). How the Pink or Blue^®^ DNA Gender Test Works. October.

[39] Ames J.J., Contois L., Caron J.M., Tweedie E., Yang X., Friesel R., Vary C., Brooks P.C. (2016). Identification of an endogenously generated cryptic collagen epitope (XL313) that may selectively regulate angiogenesis by an integrin yes-associated protein (YAP) mechano-transduction pathway. J. Biol. Chem..

[40] Emsley J., Knight C.G., Farndale R.W., Barnes M.J., Liddington R.C. (2000). Structural Basis of Collagen Recognition by Integrin α2β1. Cell.

[41] Knight C.G., Morton L.F., Peachey A.R., Tuckwell D.S., Farndale R.W., Barnes M.J. (2000). The collagen-binding a-domains of integrins α1/β1 and α2/β1 recognize the same specific amino acid sequence, GFOGER, in native (triple- helical) collagens. J. Biol. Chem..

[42] Davidenko N., Schuster C.F., Bax D.V., Farndale R.W., Hamaia S., Best S.M., Cameron R.E. (2016). Evaluation of cell binding to collagen and gelatin: A study of the effect of 2D and 3D architecture and surface chemistry. J. Mater. Sci. Mater. Med..

[43] Luo B.-H., Carman C.V., Springer T.A. (2007). Structural Basis of Integrin Regulation and Signaling. Annu. Rev. Immunol..

[44] Schmidt J.A., Rinaldi S., Scalbert A., Ferrari P., Achaintre D., Gunter M.J., Appleby P.N., Key T.J., Travis R.C. (2016). Plasma concentrations and intakes of amino acids in male meat-eaters, fish-eaters, vegetarians and vegans: A cross-sectional analysis in the EPIC-Oxford cohort. Eur. J. Clin. Nutr..

[45] To W.S., Midwood K.S. (2011). Plasma and cellular fibronectin: Distinct and independent functions during tissue repair. Fibrogen. Tissue Rep..

[46] Sparrow R.L., Simpson R.J., Greening D.W. (2017). A Protocol for the Preparation of Cryoprecipitate and Cryo-depleted Plasma for Proteomic Studies. Methods Mol. Biol..

[47] Nascimento B., Goodnough L.T., Levy J.H. (2014). Cryoprecipitate therapy. Brit. J. Anaesth..

[48] Hynes R.O. (2009). The extracellu. Science.

[49] Sahni A., Guo M., Sahni S.K., Francis C.W. (2004). Interleukin-1β but not IL-1α binds to fibrinogen and fibrin and has enhanced activity in the bound form. Blood.

[50] Martino M.M., Briquez P.S., Ranga A., Lutolf M.P., Hubbell J.A. (2013). Heparin-binding domain of fibrin(ogen) binds growth factors and promotes tissue repair when incorporated within a synthetic matrix. Proceedings of the National Academy of Sciences of the United States of America. Soc. Exploit. Vitellogen..

[51] Jeon O., Ryu S.H., Chung J.H., Kim B.-S. (2005). Control of basic fibroblast growth factor release from fibrin gel with heparin and concentrations of fibrinogen and thrombin. J. Control. Release.

[52] Briganti E., Spiller D., Mirtelli C., Kull S., Counoupas C., Losi P., Senesi S., Di Stefano R., Soldani G. (2010). A composite fibrin-based scaffold for controlled delivery of bioactive pro-angiogenetic growth factors. J. Control. Release.

[53] Panetti T.S., Kudryk B.J., Mosher D.F. (1999). Interaction of recombinant procollagen and properdin modules of thrombospondin-1 with heparin and fibrinogen/fibrin. J. Biol. Chem..

[54] Hadjipanayi E., Kuhn P.-H., Moog P., Bauer A.-T., Kuekrek H., Mirzoyan L., Hummel A., Kirchhoff K., Salgin B., Isenburg S. (2015). The fibrin matrix regulates angiogenic responses within the hemostatic microenvironment through biochemical control. PLoS ONE.

[55] Sadeghi-Ataabadi M., Mostafavi-pour Z., Vojdani Z., Sani M., Latifi M., Talaei-Khozani T. (2017). Fabrication and characterization of platelet-rich plasma scaffolds for tissue engineering applications. Mater. Sci. Eng..

[56] Loh Q.L., Choong C. (2013). Three-Dimensional Scaffolds for Tissue Engineering Applications: Role of Porosity and Pore Size. Tissue Eng. Part B Rev..

[57] Fan C., Wang D.-A. (2017). Macroporous Hydrogel Scaffolds for Three-Dimensional Cell Culture and Tissue Engineering. Tissue Eng. Part B Rev..

[58] Caliari S.R., Burdick J.A. (2016). A practical guide to hydrogels for cell culture. Nat. Methods.

[59] Hohmann T., Dehghani F. (2019). The Cytoskeleton—A Complex Interacting Meshwork. Cells.

[60] Tang D.D., Gerlach B.D. (2017). The roles and regulation of the actin cytoskeleton, intermediate filaments and microtubules in smooth muscle cell migration. Resp. Res..

[61] Martino F., Perestrelo A.R., Vinarský V., Pagliari S., Forte G. (2018). Cellular mechanotransduction: From tension to function. Front. Physiol..

[62] Chen W., Sun Y., Fu J. (2013). Microfabricated Nanotopological Surfaces for Study of Adhesion-dependent Cell mechanosensitivity. Small.

[63] Argentati C., Morena F., Tortorella I., Bazzucchi M., Porcellati S., Emiliani C., Martino S. (2019). Insight into mechanobiology: How stem cells feel mechanical forces and orchestrate biological functions. Int. J. Sci..

[64] Shannalee R., Maresha S.M., Gay L.Z. (2016). Revisiting the matricellular concept. Physiol. Behav..

[65] Aleynik D.Y., Zagaynova E.V., Egorikhina M.N., Charykova I.N., Rogovaya O.S., Rubtsova Y.P., Popova A.N., Vorotelyak E.A. (2019). Methods for Assessing the Quality of Biomedical Cell Products for Skin Replacement. Sovremennye Tehnologii v Med..

[66] Rodríguez J.P., González M., Ríos S., Cambiazo V. (2004). Cytoskeletal organization of human mesenchymal stem cells (MSC) changes during their osteogenic differentiation. J. Cell. Biochem..

[67] Saidova A.A., Vorobjev I.A. (2020). Lineage Commitment, Signaling Pathways, and the Cytoskeleton Systems in Mesenchymal Stem Cells. Tissue Eng. Part B Rev..

[68] Alonso J.L., Goldmann W.H. (2016). Cellular mechanotransduction. AIMS Biophys..

[69] Karaman S., Leppänen V.M., Alitalo K. (2018). Vascular endothelial growth factor signaling in development and disease. Development.

[70] Nasser M., Wu Y., Danaoui Y., Ghosh G. (2019). Engineering microenvironments towards harnessing pro-angiogenic potential of mesenchymal stem cells. Mater. Sci. Eng. C.

[71] Assi R., Foster T.R., He H., Stamati K., Bai H., Huang Y., Hyder F., Rothman D., Shu C., Homer-Vanniasinkam S. (2016). Delivery of mesenchymal stem cells in biomimetic engineered scaffolds promotes healing of diabetic ulcers. Regen. Med..

[72] Spees J.L., Lee R.H., Gregory C.A. (2016). Mechanisms of mesenchymal stem/stromal cell function. Stem Cell Res. Therapy.

